# $M_W$ helps select $Z^\prime$ models for $b\rightarrow s \ell \ell$ anomalies

**DOI:** 10.1140/epjc/s10052-022-10693-3

**Published:** 2022-08-24

**Authors:** Ben Allanach, Joe Davighi

**Affiliations:** 1 DAMTP, University of Cambridge, Wilberforce Road, Cambridge, CB3 0WA UK; 2 Physik-Institut, Universität Zürich, 8057 Zurich, Switzerland

## Abstract

As shown in Allanach et al. (Global fits of third family hypercharge models to neutral current B-anomalies and electroweak precision observables. arXiv:2103.12056), the Third Family Hypercharge ($Y_3$) Model changes the Standard Model prediction for $M_W$ whilst simultaneously explaining anomalies in $b\rightarrow s\ell \ell$ transitions via a heavy $Z^\prime$ gauge boson which is spawned by a spontaneously broken gauged $U(1)_{Y_3}$ symmetry. The 2022 CDF II measurement of $M_W$, which is far from the Standard Model prediction in the statistical sense, somewhat disfavours the $Y_3$ model. Here, we generalise the gauge charge assignments to the anomaly-free combination $s Y_3 + t (B_3-L_3)$ and show that incorporating the 2022 CDF II measurement of $M_W$ selects a viable domain of integers *s* and *t*. For example, $s=1, t=-3$ yields a *p* value of .08 in a two-parameter global fit to 277 electroweak and flavour changing *b* data, much improving a SM *p* value of $1\times 10^{-6}$.

## Introduction

Recently, the CDF II Collaboration reported a measurement of the *W* boson mass $M_W=80.4335\pm 0.0094$ GeV [2] that disagrees by many sigma with the Standard Model (SM) prediction of the quantity. One average^1^ of all current measurements yields [4]

1.1 $$\begin{aligned} M_W = 80.4133 \pm 0.0080 \text {~GeV}, \end{aligned}$$

still in significant disagreement (with a pull of $-6.4\sigma$) with SM predictions; we call this disagreement the $M_W$ anomaly. Note that the top quark mass $m_t$ plays an important role in the prediction of $M_W$ in the SM: throughout this paper, we impose the following constraint upon the top quark pole mass,

1.2 $$\begin{aligned} m_t=171.79 \pm 0.38 \text {~GeV}, \end{aligned}$$

based on a combination of the latest data [4]. We use the smelli2.3.2 defaults [5] for other electroweak constraints and parameters.

There are also discrepancies between SM predictions and some measurements of flavour-changing *B*-meson decays, which we collectively call the $b\rightarrow s\ell \ell$ anomalies. For example, various lepton flavour universality (LFU) observables like the ratios of branching ratios $R_{K^{(*)}}=BR(B \rightarrow K^{(*)} \mu ^+ \mu ^-)/BR(B \rightarrow K^{(*)} e^+ e^-)$ are observed to be lower than their SM predictions in several channels and several different bins of di-lepton invariant mass squared [6–8]. Similar double ratio measurements in $B^0 \rightarrow K^0_s \ell ^+ \ell ^-$ and $B^+ \rightarrow K^{*+} \ell ^+ \ell ^-$ decays [9] are consistent with a similar deficit in di-muons over di-electrons, albeit with lower statistics than in $R_K^{(*)}$. The predictions for all aforementioned double ratios have rather small theoretical uncertainties due to cancellations and the SM predictions are generically considered to be robust in the di-lepton invariant mass squared bins of interest. $BR(B_s\rightarrow \mu ^+\mu ^-)$ also has quite small theoretical uncertainties in its SM prediction. The combined measurements of $BR(B_s\rightarrow \mu ^+\mu ^-)$ are in a 2$\sigma$ tension with its SM prediction [10–14]. Several other *B*-meson decay observables appear to be in tension with SM predictions even when their larger theoretical uncertainties are taken into account: for example angular distributions in $B\rightarrow K^*\mu ^+ \mu ^-$ decays [15–20], and $BR(B_s \rightarrow \phi \mu ^+ \mu ^-)$ [21, 22]. For these quantities though, there is room for argument about the assumed size of the theoretical uncertainties and indeed the best way of estimating the SM predictions for them.

**Table 1:** SM goodness of fit as calculated by smelli2.3.2. $n_{obs}$ shows the number of observables in each category.

Category	$n_{obs}$	$\chi ^2$	*p*	*p* (global)	Pull ($M_W$)
Quarks	224	269	.021		
LFU FCNCs	23	39.4	.018		
EWPOs (smelli)	31 s	38.4	.17	.0036	$-2.1$
EWPOs (CDF II)	30	93.6	2.9$\times 10^{-6}$	$1.3\times 10^{-6}$	$-6.4$

$\chi ^2$ denotes the $\chi ^2$ statistic within that category, *p* is the *p* value of the category, and *p*(global) is the global *p* value of all observables. The category ‘LFU FCNCs’ contains lepton flavour universality violating flavour changing observables such as $R_K^{(*)}$ as well as $BR(B_s \rightarrow \mu ^+ \mu ^-)$, where theoretical uncertainties are relatively small. The ‘quarks’ category contains other flavour-changing *b* observables, some of which have large theoretical uncertainties. The category EWPOs (smelli) contains electroweak precision observables for the default smelli2.3.2 combination of $M_W$ (4.13) which excludes the CDF II measurement, whereas EWPOs(CDF II) includes it in a global average á la (1.1), (1.2). *p*(global) includes the ‘quarks’ category, the ‘LFU FCNCs’ category and the ‘EWPO’ category relevant for the respective combination of $M_W$ measurements. For a definition of the pull, see (4.14) and the discussion of it in the surrounding text

We display the statistical tensions in the SM due to the $M_W$ and $b\rightarrow s \ell \ell$ anomalies in Table 1, as calculated by the computer program^2^smelli2.3.2 [5]. We see from the table that the $b\rightarrow s \ell \ell$ anomalies disfavour the SM: the $b\rightarrow s \ell \ell$ data yields a poor fit with a global *p* value of .0036, even when using the default smelli2.3.2 constraints upon the EWPOs (4.13), i.e. *excluding* the new CDF II measurement of $M_W$ (although the EWPOs have an acceptable fit in and of themselves with a *p* value of .17). Taking into account the CDF II measurement of $M_W$ as in (1.1) exacerbates an already poor quality of fit, lowering the global *p* value to $10^{-6}$.

Global fits find that new physics contributions to the $(\overline{b} \gamma ^\mu P_L s) (\overline{\mu } \gamma _\mu P_X \mu )+H.c.$ vertex in the Lagrangian density can ameliorate the fit to the $b\rightarrow s \ell \ell$ data [23–29], where $P_L$ is the left-handed projection operator. $P_X$ corresponds to the helicity projection of the muon pair in the effective vertex: the fits agree that a purely right-handed projection $P_X=P_R$ is disfavoured, whereas a mixture in the domain $P_X\approx P_L$ to $P_X\approx P_L+P_R$ is preferred [30]. Whereas the fitting groups yield very similar results when fitting just to the ‘LFU’ category of observables, there are some differences observed when the ‘quarks’ category is included: for example, which option out of $P_X=P_L$ or $P_X=P_L+P_R$ has a better fit. Such differences in the constraints can arise from the treatment of theoretical uncertainties in the ‘quarks’ category. The lesson one learns from such studies is that, from a new physics point of view in order to fit the $b\rightarrow s \ell \ell$ anomalies, one needs a new physics state that couples to left-handed quark fields and left-handed muon fields in a family non-universal manner (but it may or may not also couple to right-handed muon fields and/or electron fields). According to Ref. [31], there is a mild preference for a family universal coupling of new physics to leptons as well as a specific and different new physics contribution to the coupling to di-muon pairs.

One category of new physics state that can have such couplings is a heavy electrically-neutral vector boson, dubbed a $Z^\prime$ [32–36]. In specific models, one obtains the $Z^\prime$ from a spontaneously broken additional $U(1)_X$ gauge symmetry under which the SM fermions have family dependent charges. In a consistent ultra-violet (UV) complete model, quantum field theoretic anomalies should cancel.^3^ Our model here will not be UV complete, since new physics above the TeV scale is required to generate the light Yukawa couplings (once integrated out and matched onto our SM$\times U(1)_X$ model). Nevertheless, it is wise to cancel gauge anomalies in the SM$\times U(1)_X$ model, as we do here; if not, there must be heavy chiral fermions to cancel anomalies, whose masses are at least tied to the TeV-scale $U(1)_X$ breaking. Many viable anomaly-free $U(1)_X$ charge assignments have been investigated: for example muon minus tau lepton number [41–46], third family baryon number minus second family lepton number [47–49], third family hypercharge [50–52] or other assignments [53–72].

In Ref. [50], the Third Family Hypercharge ($Y_3$) model was presented and shown to fit the $b\rightarrow s \ell \ell$ data. Here, the *X* charges of all fermions are zero except for the third family, which has *X* charge equal to its hypercharge. The model predicts $Z-Z^\prime$ mixing because the Higgs doublet is necessarily charged under $U(1)_X$ to allow a renormalisable top Yukawa coupling, which would otherwise be forbidden by the $U(1)_X$ gauge symmetry. It was noted that this will change the SM prediction for $M_W$ [73] and in Ref. [1] it was shown that the model could simultaneously fit the $b\rightarrow s \ell \ell$ anomalies and EWPOs: indeed, that the SM prediction for $M_W$ was improved, since it was some $2 \sigma$ too low in the SM (compared to the measurement at the time) but receiving a positive correction from the $Y_3$ model.

We will show below that including the new CDF II measurement of $M_W$ as in (1.1), the $Y_3$ model is somewhat disfavoured with a global *p* value of .02. Our purpose in this paper is then to build a generalisation of the $Y_3$ model, which is similar in construction and as simple, but which provides a simultaneously acceptable fit to both the $M_W$ anomaly and $b\rightarrow s \ell \ell$ anomalies. To this end, in Sect. 2, we shall propose a generalisation of the $Y_3$ charge assignments, which is rendered anomaly-free simply by the inclusion of right-handed neutrinos. There are two classes of charge assignment, depending upon whether one permutes the *right-handed* third family field’s charged lepton charge with that of the second family (i.e. $X_{e_3} \leftrightarrow X_{e_2}$) or not. In Sect. 3, we calculate the SM effective field theory (SMEFT) coefficients that arise from integrating out the heavy $Z^\prime$ boson, and then matching to the SMEFT at tree level. This allows us to encode our model in a suitable form for input into smelli2.3.2 and use its calculation of EWPOs, LFU observables and the ‘quarks’ category of observables. We discuss the phenomenology of the models in Sect. 4, in particular the effect on $M_W$ and on $b\rightarrow s \ell \ell$ coefficients in the weak effective theory. The case where one permutes $X_{e_3} \leftrightarrow X_{e_2}$ corresponds to $P_X=P_L+P_R$, whereas in the case where one does not, one obtains a certain linear combination of $P_L$ and $P_R$, depending upon *s* and *t*. In both cases, there is a purely axial coupling to electrons. We then present global fits to this latter family of model. The inclusion of the 2022 CDF II $M_W$ measurement selects a subset of models which provide acceptable fits to the collective data. We conclude in §5.

## The models

We consider a class of $Z^\prime$ models based on extending the SM gauge symmetry by a $U(1)_X$ factor, where

2.1 $$\begin{aligned} X = s Y_3 + t (B-L)_3\, , \qquad s \in \mathbb {N},\, t \in \mathbb {Z}, \end{aligned}$$

$Y_3$ is third family hypercharge and $(B-L)_3$ is third family baryon number minus lepton number. Our conventions for the representations of the non-gauge fields in the model are shown in Table 2.

**Table 2:** Representations of fields under the SM gauge factors, which are family universal, together with the family non-universal $Y_3$ and $(B-L)_3$ symmetries on which our $Z^\prime$ model is based.

	$q_{1}^{\prime }$ $q_{2}^{\prime }$ $q_{3}^{\prime }$	$u_{1}^{\prime }$ $u_{2}^{\prime }$ $u_{3}^{\prime }$	$d_{1}^{\prime }$ $d_{2}^{\prime }$ $d_{3}^{\prime }$	$\ell _{1}^{\prime }$ $\ell _{2}^{\prime }$ $\ell _{3}^{\prime }$	$e_{1}^{\prime }$ $e_{2}^{\prime }$ $e_{3}^{\prime }$	$\nu _{1}^{\prime }$ $\nu _{2}^{\prime }$ $\nu _{3}^{\prime }$	*H*	$\theta$
SU(3)	**3**	**3**	**3**	**1**	**1**	**1**	**1**	**1**
SU(2)	**2**	**1**	**1**	**2**	**1**	**1**	**2**	**1**
$U(1)_Y$	1	4	$-2$	$-3$	$-6$	0	3	0
$U(1)_{Y_3}$	0 0 1	0 0 4	0 0 $-2$	0 0 $-3$	0 0 $-6$	0 0 0	3	$*$
$U(1)_{(B-L)_3}$	0 0 1	0 0 1	0 0 1	0 0 $-3$	0 0 $-3$	0 0 $-3$	0	$*$

We use the minimal integer normalisation for the charges under each *U*(1) factor. Note that the permutations $\ell _2 \leftrightarrow \ell _3$ (and in some cases $e_2 \leftrightarrow e_3$) are made *after* the assignments shown. All fields are Weyl fermions except for the complex scalar Higgs doublet *H* and the complex scalar flavon $\theta$.$*$ denotes that the charge is a non-zero number whose value does not change any of the discussion or results of this paper

The *X* charge assignment in (2.1) is the most general anomaly-free $U(1)_X$ extension^4^ that couples only to a single family of SM fermions [38], including a right-handed neutrino. While we have parameterised this family of $U(1)_X$ models by two integers *s* and *t*, it is only the rational parameter $t/s \in {\mathbb {Q}}$ that is relevant for phenomenology.^5^ This family of $U(1)_X$ extensions of the SM are known to have semi-simple gauge completions without needing any extra chiral fermions, as was shown in Ref. [74].

The $Z^\prime$ model is designed to explain the $b\rightarrow s\ell \ell$ anomalies. Global fits strongly favour a lepton flavour non-universal coupling of the $Z^\prime$ to left-handed muons, at least, as described in Sect. 1. We thus permute the non-zero left-handed lepton charge from the third to the second family, as in the original $Y_3$ model of [50]. Regarding right-handed leptons, acceptable fits can be obtained with or without permuting the non-zero right-handed lepton charge to the second family.

In summary, the charges of the SM fermions, together with the SM Higgs doublet $H=(H^+,H^0)^T$ and an extra $U(1)_X$ symmetry-breaking scalar $\theta$ which is a SM singlet, are

2.2 $$\begin{aligned} X_{q_3}&= s+t, \quad X_{\ell _2} = -3s-3t, \end{aligned}$$

2.3 $$\begin{aligned} X_{u_3}&= 4s+t, \quad X_{e_n} = -6s-3t,\quad n=2\text {~or~}3\,,\end{aligned}$$

2.4 $$\begin{aligned} X_{d_3}&= -2s+t, \quad X_{\nu _n} = -3t, \end{aligned}$$

2.5 $$\begin{aligned} X_{H}&=3s, \quad X_{\theta } , \end{aligned}$$

where $X_\theta \ne 0$, with all other $U(1)_X$ charges being zero. The original $Y_3$ model of Ref. [50], which is a template for the family of models we consider here, can be recovered by setting $n=3$ and $(s,t)=(1,0)$, thus decoupling the right-handed neutrino.

Like the $Y_3$ model [50], the family of models we consider here allow, on the quark side, only third family Yukawa couplings to the Higgs at the renormalisable level, $\mathcal {L} \supset y_t \overline{q}_3^\prime H^c u_3^\prime + y_b \overline{q}_3^\prime H d_3^\prime$. Thus, to zeroth order, gauging the anomaly-free chiral third-family symmetry (2.5) postdicts a heavy third family and small quark mixing angles, as observed. The light quark Yukawa couplings, responsible for the masses of the first and second generation and for the quark mixing angles, must come from higher-dimensional operators. Such operators can come from integrating out heavier fermionic representations that are vector-like under to the gauge group. These are details of the UV theory which it would be premature to specify (although see Ref. [1] for further comments and ideas).

On the other hand, the lepton sector is not so natural from the Yukawa perspective, due to the fact that we permute the non-zero $X_{\ell _i}$ charge into the second family. At face value, the charge assignment (2.5) with $n=3$ allows only for a renormalisable off-diagonal Yukawa coupling $\sim \overline{\ell }_2^\prime H e_3^\prime$, which must be highly suppressed in order to explain the relative heaviness of the tau and the non-observation of $\mu -\tau$ lepton flavour violation. This is easily explained with a little more model-building; for example, additionally gauging an anomaly-free lepton-flavoured *U*(1) symmetry could ban this off-diagonal Yukawa coupling.^6^

In what follows we use a convention in which the covariant derivative acting on a field *f* is

2.6 $$\begin{aligned} D_\mu = \partial _\mu - i g \frac{\sigma ^a}{2} P_L W_\mu ^a - i g^\prime Y_f B_\mu - i g_X X_f X_\mu , \end{aligned}$$

where $X_\mu$ is the gauge field for $U(1)_X$ and $g_X$ is its gauge coupling. We assume that any kinetic mixing between $U(1)_X$ and $U(1)_Y$ gauge fields is negligible, leaving a study of such effects to future work. The fermion couplings to the gauge fields, in the gauge eigenbasis (indicated by ‘primes’), are

2.7 $$\begin{aligned} \mathcal {L}_{\psi } = g_X X_\mu \sum _{\psi } X_{\psi } \bar{\psi }^\prime \gamma ^\mu \psi ^\prime , \end{aligned}$$

where the sum on $\psi$ runs over all SM Weyl fermions and the charges $X_\psi$ are those in (2.5).

### Symmetry breaking and ${Z-Z}^\prime$ mixing

The $U(1)_X$ symmetry is broken predominantly by the SM singlet scalar field $\theta$, which acquires a vacuum expectation value $\langle \theta \rangle = v_X/\sqrt{2}$, where $v_X \gg v$ is of order the TeV scale. This implies that the mass of the $Z^\prime$ field $M_{Z^\prime } \approx g_X X_\theta v_X$. However, because the SM Higgs is also charged under $U(1)_X$, there is mass mixing between the *Z* boson and the $Z^\prime$ gauge boson. These physical gauge bosons are linear combinations of the SM combination^7^$Z^0_\mu = c_w W^3_\mu - s_w B_\mu$ (where $\theta _w = \text {tan}^{-1}(g'/g)$ is the Weinberg angle) and the $X_\mu$ field viz.

2.8 $$\begin{aligned} \begin{pmatrix} Z_\mu \\ Z^\prime _\mu \end{pmatrix} = \begin{pmatrix} c_z &{} s_z \\ -s_z &{} c_z \end{pmatrix} \begin{pmatrix} Z^0_\mu \\ X_\mu \end{pmatrix}, \end{aligned}$$

where the mixing angle $\alpha _z$ is determined by, at tree-level,

2.9 $$\begin{aligned} \sin \alpha _z = \frac{X_H}{2X_\theta ^2} \frac{g/c_w}{g_X} \frac{v^2}{v_X^2}= \frac{2X_H g_X}{g/c_w}\frac{M_Z^2}{M_{Z^\prime }^2} \left[ 1 + \mathcal {O}\left( \frac{M_Z^2}{M_{Z^\prime }^2}\right) \right] , \end{aligned}$$

where

2.10 $$\begin{aligned} M_Z^2 = v^2 g^2/4c_w^2 \left[ 1 + \mathcal {O}\left( \frac{M_Z^2}{M_{Z^\prime }^2}\right) \right] . \end{aligned}$$

At a constant value of $M_Z$ (we shall take $M_Z$ to be an experimental input), the ${\mathcal O}(M_Z^2/M_{Z^\prime }^2)$ correction in (2.10) translates to an upward shift in the SM prediction for $M_W$, as we shall describe in Sect. 4.1.

### Fermion mixing matrices

Following Refs. [1, 50], we assume a simple ansatz for the 3-by-3 unitary fermion mixing matrices describing the change from the gauge eigenbasis to the (unprimed) mass eigenbasis of the fermionic fields. The purpose of this ansatz is to characterise the main physical flavour characteristics of the model without introducing large flavour-changing neutral currents that would be subject to strong experimental constraints. We may think of the ansatz as a limit to expand around: the fairly strong assumptions might be motivated by further model-building involving additional symmetries or dynamics, but we leave such considerations to one side, for now.

For left-handed down-type quarks, we parameterise the mixing matrix as

2.11 $$\begin{aligned} V_{d_L}= \left( \begin{array}{ccc} 1 &{} 0 &{} 0 \\ 0 &{} \cos \theta _{sb} &{} \sin \theta _{sb} \\ 0 &{} -\sin \theta _{sb} &{} \cos \theta _{sb} \\ \end{array} \right) . \end{aligned}$$

For simplicity, and to avoid unwanted large contributions to $\Delta F = 2$ processes and charged lepton flavour violating processes, we choose $V_{d_R}=1$, $V_{u_R}=1$ and $V_{e_L}=1$. Finally, $V_{u_L} = V_{d_L}V^\dagger$ and $V_{\nu _L} = V_{e_L}U^\dagger$ are fixed by the CKM matrix *V* and the PMNS matrix *U*, respectively. We use the central values of extracted angles and phases from the Particle Data Group [75].

Using these rotation matrices, the couplings of the $Z^\prime$ boson to the physical fermion states can be obtained from (2.7). For the left-handed quarks, we work in a ‘down-aligned’ basis in which the (unprimed) quark doublets are $q_i = (V^\dagger _{ij} u_{L,j},\ ~d_{L,i})$. For the left-handed leptons, we work in a basis in which the left-handed charged leptons $e_{L_i}$ align with the mass eigenstates, and thus the (unprimed) lepton doublets are $\ell _i = (U^\dag _{ij} \nu _{L,j},\ ~e_{L, i})$. Since $V_{e_L} = V_{e_R}=1$ the charged lepton couplings remain diagonal. The down quark couplings are mixed away from the diagonal, however, as is indeed necessary to obtain a quark flavour-violating coupling of the $Z^\prime$ to $b\bar{s}$ and $\bar{s}b$. We have:

2.12 $$\begin{aligned}&\mathcal {L}_{\psi } \supset g_X X_\mu \big ( X_{q_3}\Lambda ^{d_L}_{ij} \overline{q}_i \gamma ^\mu q_j + X_{u_3} \overline{u}_3 \gamma ^\mu u_3 + X_{d_3} \overline{d}_3 \gamma ^\mu d_3 \nonumber \\&\quad + X_{\ell _2} \overline{\ell }_2 \gamma ^\mu \ell _2 + X_{e_n} \overline{e}_n \gamma ^\mu e_n + X_{\nu _n} \overline{\nu }_n \gamma ^\mu \nu _n \big ) , \end{aligned}$$

where $$\Lambda ^{d_L}_{ij} := V_{d_L}^\dagger \text {diag}(0,0,1) V_{d_L}$$.

## SMEFT matching

Integrating out the heavy *X* boson and matching onto the SM Effective Field Theory (SMEFT) at a scale $M_X:=g_X X_\theta v_X$, we obtain the Wilson coefficients (WCs) for dimension-6 SMEFT operators written in Table 3. The WCs $\{C_i\}$ have units of $[\text {mass}]^{-2}$ and are written in the Warsaw basis [76], a basis of a set of independent baryon-number conserving operators. By performing the matching between our models and the SMEFT, we obtain the set of WCs $\{C_i\}$ at the scale $M_X$, which can then be used to calculate predictions for observables.

**Table 3:** Non-zero dimension-6 SMEFT Wilson coefficients in the Warsaw basis, obtained by integrating out the heavy *X* boson at scale $M_{X}$.

WC	Value	WC	Value
$$C_{ll}^{2222}$$	$$-\frac{1}{2} X_{\ell _2}^2$$	$$ (C_{lq}^{(1)})^{22ij}$$	$$-X_{q_3}X_{\ell _2} \Lambda _{ij}^{d_L}$$
$(C_{qq}^{(1)})^{ijkl}$	$X_{q_3}^2{\Lambda _{ij}^{d_L}} {\Lambda _{kl}^{d_L}}\frac{\delta _{ik}\delta _{jl}-2}{2}$	$C_{ee}^{nnnn}$	$-\frac{1}{2} X_{e_n}^2$
$C_{uu}^{3333}$	$-\frac{1}{2} X_{u_3}^2$	$C_{dd}^{3333}$	$-\frac{1}{2} X_{d_3}^2$
$C_{eu}^{nn33}$	$-X_{e_n}X_{u_3}$	$C_{ed}^{nn33}$	$-X_{e_3}X_{d_3}$
$(C_{ud}^{(1)})^{3333}$	$-X_{u_3}X_{d_3}$	$C_{le}^{22nn}$	$-X_{\ell _2}X_{e_n}$
$C_{lu}^{2233}$	$-X_{\ell _2}X_{u_3}$	$C_{ld}^{2233}$	$-X_{\ell _2}X_{d_3}$
$C_{qe}^{ijnn}$	$-X_{q_3}X_{e_n} \Lambda _{ij}^{d_L}$	$(C_{qu}^{(1)})^{ij33}$	$-X_{q_3}X_{u_3} \Lambda _{ij}^{d_L}$
$(C_{qd}^{(1)})^{ij33}$	$-X_{q_3}X_{d_3} \Lambda _{ij}^{d_L}$	$(C_{\phi l}^{(1)})^{22}$	$-X_H X_{\ell _2}$
$(C_{\phi q}^{(1)})^{ij}$	$-X_H X_{q_3}$	$C_{\phi e}^{nn}$	$-X_H X_{e_n}$
$C_{\phi u}^{33}$	$-X_H X_{u_3}$	$C_{\phi d}^{33}$	$-X_H X_{d_3}$
$C_{\phi D}$	$-2 X_H^2$	$C_{\phi \Box }$	$-\frac{1}{2} X_H^2$

We write the coefficients as functions of the charges $X_f$, which are explicitly parameterised in (2.5). The integer $n=2$ or $n=3$ corresponds to two variations of the model, as explained in Sect. 2. All Wilson coefficients are in units of $g_X^2 / M_X^2$

## Phenomenology

Starting from the SMEFT matching of Sect. 3, we will use the smelli2.3.2 program to evaluate the likelihood of the model given hundreds of observables in the electroweak and flavour sectors. Before we do so, however, we think it important to highlight the most important observables that are sensitive to our model, and how these depend upon the SMEFT coefficients and hence upon the $X_f$ charges.

### Important observables

#### Electroweak

In light of the recent CDF II measurement [2], the $M_W$ prediction is deserving of special attention. We can parameterise its deviation from the SM prediction in our $Z^\prime$ model via the parameter

4.1 $$\begin{aligned} \rho _0 := \frac{M_W^2}{M_Z^2 \hat{c}_Z^2 \hat{\rho }}, \end{aligned}$$

where the parameter $\hat{\rho }=1.01019\pm 0.00009$ includes the custodial-violating top contributions to the gauge boson masses (see Ref. [75]). In (4.1), we use the conventional notation $\hat{c}_Z^2 := \cos ^2 \hat{\theta }_w(M_Z)=\frac{\hat{g}^2(M_Z)}{\hat{g}^2(M_Z)+\hat{g}'^2(M_Z)_{}}$ to denote the cosine squared of the renormalised Weinberg angle in the $\overline{\text {MS}}$ scheme. The $\rho _0$ parameter is defined so as to equal precisely 1 in the SM using the $\overline{\text {MS}}$ scheme. Its deviation from unity in our heavy $Z^\prime$ model, which recall is due to $Z-Z^\prime$ mixing, is

4.2 $$\begin{aligned} \rho _0 \approx 1 + \frac{4X_H^2 g_X^2}{g^2+g^{\prime 2}} \frac{M_Z^2}{M_{Z^\prime }^2}\, . \end{aligned}$$

Importantly, $\rho _0$ unavoidably shifts *upwards* [1, 77], easing the tension due to the CDF II $M_W$ measurement irrespective of the sign of the Higgs charge $X_H$.

At the level of the SMEFT, this shift is captured by the Wilson coefficients $C_{\phi D}$ and $C_{\phi \Box }$, which are (as in Table 3)

4.3 $$\begin{aligned} C_{\phi D} = 4 C_{\phi \Box } = -2X_H^2 \frac{g_X^2}{M_X^2} = -18s^2 \frac{g_X^2}{M_X^2}. \end{aligned}$$

Global fits of the SMEFT to electroweak data that turn on only these two operators have been found to give a good fit to electroweak data in light of the CDF measurement (e.g. [78]).

The status of the electroweak fit is of course much more delicate in our model for the $b\rightarrow s\ell \ell$ anomalies, because we have a plethora of new physics effects in *Z* pole observables due to modified *Z* couplings to fermions. Important among these are modified *Z* couplings to leptons, especially muons, and the forward-backward asymmetry variable $A^{FB}_b$ for *Z* decays to $b\bar{b}$, because these observables already exhibit small tensions with the SM and because they receive large-ish corrections in our model. These effects are strictly correlated to the shifts in $C_{\phi D}$ and $C_{\phi \Box }$, since they arise from the $Z-Z^\prime$ mixing.

#### $b\rightarrow s\ell \ell$

Regarding the $b\rightarrow s\ell \ell$ anomalies and related observables, the new physics effects can be parameterised by contributions to Wilson coefficients in the weak effective theory (WET). The WET Hamiltonian is, using a conventional normalisation,

4.4 $$\begin{aligned} \mathcal {H}_{\text {EFT}} = - \frac{4G_F}{\sqrt{2}} V_{tb} V_{ts}^*\sum _i (C_i^{\text {SM}} + C_i) \mathcal {O}_i, \end{aligned}$$

where we emphasise that in the present paper, $C_i$ denotes the *beyond* the SM (BSM) contribution to the Wilson coefficient. When coupling to $\bar{b}s$/$\bar{s}b$ currents, the $Z^\prime$ only couples to the left-handed component and so there are contributions to the dimension-6 semi-leptonic operators

4.5 $$\begin{aligned} \mathcal {O}_9^{\ell \ell }&= \frac{e^2}{16\pi ^2} (\bar{s} \gamma _\mu P_L b)(\bar{l}\gamma ^\mu \ell ) , \quad \ell \in \{e,\mu \},\nonumber \\ \mathcal {O}_{10}^{\ell \ell }&= \frac{e^2}{16\pi ^2} (\bar{s} \gamma _\mu P_L b)(\bar{l}\gamma ^\mu \gamma _5 \ell ) . \end{aligned}$$

Because of the $Z-Z^\prime$ mixing, the *Z* boson picks up a small quark flavour-violating coupling to $\bar{b}s$, *viz.* $\mathcal {L} \supset g_Z^{sb} Z_\mu \bar{b}_L \gamma ^\mu s_L+H.c.$, where the coupling $g_Z^{sb}$ is proportional to the $Z-Z^\prime$ mixing angle (2.9). Specifically,

4.6 $$\begin{aligned} g_Z^{sb} = X_{q_3} X_H \sin 2\theta _{sb} \frac{g_X^2}{g/c_w} \frac{M_Z^2}{M_Z'^2}. \end{aligned}$$

There are thus BSM contributions to the 4-fermion operators $\mathcal {O}_{9,10}^{\ell \ell }$ from both *Z* exchange and from $Z^\prime$ exchange, and these are of the same order. The *Z* contributions are lepton flavour universal (LFU), whereas the $Z^\prime$ contributions introduce lepton flavour universality violation (LFUV).

Accounting for both contributions^8^ we have

4.9 $$\begin{aligned}&C_{9}^{\mu \mu } = -N(X_{\ell _2}+X_{e_2}) + C_9^U, \quad \text {where~} C_9^U = NX_H/k \approx 0, \nonumber \\&C_{10}^{\mu \mu } = -N(-X_{\ell _2}+X_{e_2}) + C_{10}^U, \quad \text {where~} C_{10}^U = -NX_H , \nonumber \\&C_{9}^{ee} = C_9^U \approx 0, \nonumber \\&C_{10}^{ee} = C_{10}^U, \end{aligned}$$

where recall that $k=1/(1-4\sin ^2 \theta _w) \approx 9.23$. We have defined the common pre-factor

4.10 $$\begin{aligned} N=\frac{\sqrt{2}\pi ^2}{e^2 G_F} \frac{\sin 2\theta _{sb}}{V_{tb} V_{ts}^*}\frac{g_X^2 X_{q_3}}{M_{Z^\prime }^2}, \end{aligned}$$

where *e* is the electromagnetic gauge coupling. The *Z*-induced LFU pieces in (4.8) clearly vanish in the limit $X_H\rightarrow 0$, in which there is no $Z-Z^\prime$ mixing. The LFU contributions to the $b \rightarrow s \ell \ell$ observables are therefore correlated to their effects on EWPOs, including the shift in $M_W$. This correlation was explored, prior to the updated CDF II measurement, in Ref. [77].

Substituting in the parametrisation (2.2–2.5) of charges in our models, and dropping the 1/*k* suppressed $C_9^U$ contributions, we have

4.7 $$\begin{aligned} n=3: \qquad C_{9}^{\mu \mu }&\approx {3N} (-s-t) , \nonumber \\ C_{10}^{\mu \mu }&= {3N}(2s+t) , \nonumber \\ C_{10}^{ee}&= {3N}s. \end{aligned}$$

This is equivalent to writing the chirality of the coupling to muons in terms of left and right projection operators as in Sect. 1, i.e. via an effective operator $\propto (\bar{s} \gamma _\mu P_L b)(\bar{\mu } \gamma ^\mu P_X \mu )$. For a general *s* and *t*, this projection operator is

4.11 $$\begin{aligned} P_X = P_L - \frac{s}{3s+2t}P_R\, \quad (n=3). \end{aligned}$$

For the variation of the model with $n=2$, we have

4.12 $$\begin{aligned} n=2: \qquad C_{9}^{\mu \mu }&\approx 3N(-3s-2t) , \nonumber \\ C_{10}^{\mu \mu }&= 0 , \nonumber \\ C_{10}^{ee}&= 3Ns, \end{aligned}$$

thus $P_X \approx P_L+P_R$, a vectorial coupling of the $Z^\prime$ to $\mu ^+\mu ^-$.

### Global fits

We wrote a computer program (included in the ancillary information attached to the arXiv preprint of this paper) that uses smelli2.3.2 to calculate the likelihoods of 277 observables for our model by first calculating the WCs in Table 3 at scale $M_X$. smelli2.3.2 then renormalises the SMEFT operators down to the scale $M_Z$, where it calculates the EWPOs. It then matches at tree-level to the weak effective theory, and renormalises the resulting WCs down to the scale of the mass of the bottom quark, where the various observables pertinent to *B*-meson decays are calculated.

For a given *s* and *t* the likelihoods are a function of two effective parameters: $\alpha :=g_X \times \text {3 TeV}/M_X$ and the quark mixing angle $\theta _{sb}$. Throughout, we shall illustrate with $M_X=3$ TeV as an example (similar third-family type models with $M_X\ge 1$ TeV were not ruled out by search constraints in the parameter region where they fit the $b\rightarrow s \ell \ell$ anomalies [81, 82] and so we expect $M_X = 3$ TeV to be allowed by direct $Z^\prime$ search constraints). We do not expect a significant change if we were to change $M_X$ to a different value $M_{X^\prime }$ as long as one scales $g_X$ by the same factor, since the boundary conditions upon the Wilson coefficients (shown in Table 3) depend only upon the ratio $g_X/M_X$. This approximation is good up to small loop suppressed corrections from the renormalisation group running between $M_{X^\prime }$ and $M_{X}$, which induce relative multiplicative changes of ${\mathcal O}[\log (M_{X^\prime }/M_X)/(16 \pi ^2)]$ in corrections to predictions of observables^9^.

We consider each different value of the ratio *t*/*s* to constitute a different model. $\mathtt{smelli2.3.2}$ then calculates $\chi ^2$ via the predicted values of observables. We minimise $\chi ^2$ by varying $\alpha$ and $\theta _{sb}$ using the Nelder–Mead algorithm, given a guess at a starting point. This was obtained by doing a rough initial scan for one value of $t\ne 0$ and $s\ne 0$ and roughly estimating how electroweak and *b*-observable $\chi ^2$ values are expected to scale with *s* and *t*.

**Fig. 1:**
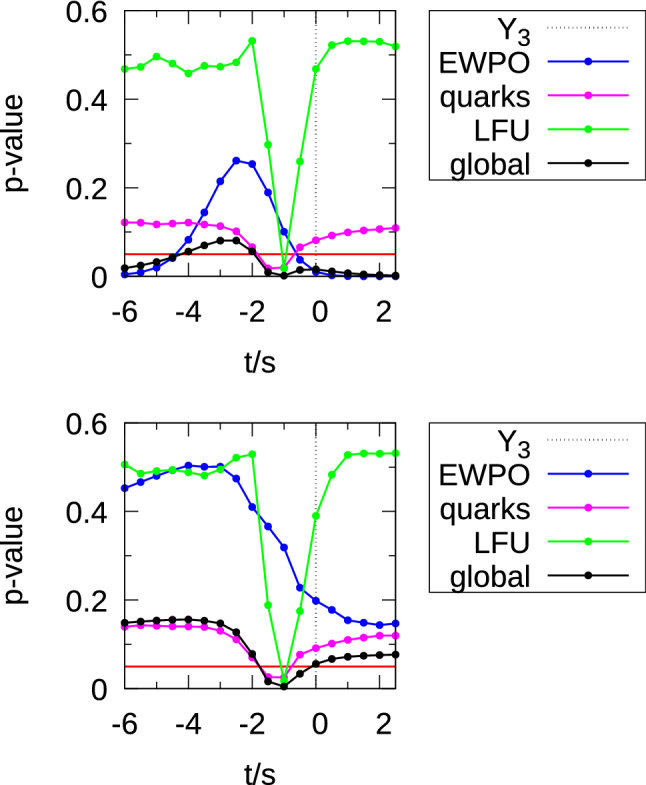
*p* values for different models depending upon *t*/*s*, for $n=3$. The top panel includes the recent CDF measurement of $M_W$ as in (1.1), whereas in the bottom panel, the default smelli2.3.2 constraint (i.e. excluding the recent CDF measurement) on $M_W$ was used as in (4.13). The Third Family Hypercharge Model is marked in the legend by $Y_3$. Only the global *p* value accounts for the 2 fitted parameters in the calculation of the number of degrees of freedom. We consider global *p* values above the red line at 0.05 to be acceptable fits to the data

The result, for a given value of *t*/*s*, is a best-fit point, where the fit in the EWPOs is balanced against those of the *b* data. This balance crucially depends upon the experimental constraint that is taken on $M_W$, as we illustrate in the top panel of Fig. 1, where we display *p* values using (1.1), which *includes* the CDF II measurement. Here, we can see that, for example, the Third Family Hypercharge Model model, denoted $Y_3$, is somewhat disfavoured: its global *p* value is around .02. Models at larger values of |*t*/*s*| approximate the $B_3-L_3$ model and so the *p* values all asymptote towards the left-hand side and the right-hand side of the plot. The $B_3-L_3$ model has no $Z-Z^\prime$ mixing because the Higgs doublet field is not charged under $B_3-L_3$, meaning that in the limit of large |*t*/*s*|, the CDF II $M_W$ measurement strongly disfavours each model. We see that models with $-5< t/s < -2$ all fare well with global *p* values above the canonical .05 bound shown by the red line. We notice by examining the EWPO *p* values that the $M_W$ constraint prefers a localised value of *t*/*s*. The other effects that are relevant are in the LFU and ‘quarks’ categories: both have a valley around $t/s=-1$. Here, at $s=-t$, one can see from (2.2) that $X_{q_3}=X_{\ell _2}=0$, meaning that there is no coupling at tree-level between the $Z^\prime$ and left-handed quarks or left-handed muons; the $Z^\prime$ does therefore not help with the $b\rightarrow s \ell \ell$ anomalies and we revert to the poor fit of the SM both for the ‘LFU’ and for the ‘quarks’ category of observable. It is of interest that the *p* value is suppressed somewhat for all *t*/*s* by the ‘quarks’ category of observable, in which there is room for disagreement with the theoretical uncertainty budget of the prediction.

We see a rather weak $M_W$ selection effect in the bottom panel of Fig. 1 for the default experimental $M_W$ constraint in smelli2.3.2, which amounts to

4.13 $$\begin{aligned} M_W=80.3795 \pm 0.0121 \text {~GeV} \end{aligned}$$

(the central value of the SM prediction according to smelli2.3.2 is $M_W=80.3509$ GeV). The smelli2.3.2 $M_W$ constraint leads to weaker selection than the one including the CDF II $M_W$ measurement because it has much larger uncertainties and less need for a large contribution from the $Z^\prime$. We see here that only the region $-2<t/s<0$ has a global *p* value of *less* than .05, and this is clearly driven by the ‘quarks’ and ‘LFU’ categories, not by EWPOs. We summarise the $\chi ^2$ and *p* values for both options of experimental $M_W$ constraint in Table 4.

**Table 4:** *p* values for $s=2, t=-6$ for the two different different constraints upon $M_W$ and $n=3$.

Category	$\chi ^2$	$n_{obs}$	*p* value
Including CDF II $M_W$ (1.1)			
$g_X\times \text {3~TeV}/M_{Z^\prime }=0.0209$, $\theta _{sb}=-0.0191$			
Quarks	246.7	224	.11
LFU FCNCs	22.8	23	.47
EWPOs	35.8	30	.21
Global	305.3	277	.08
Default smelli2.3.2 $M_W$ (4.13)			
$g_X\times \text {3~TeV}/M_{Z^\prime }=0.0150$, $\theta _{sb}=-0.0361$			
Quarks	248.7	224	.13
LFU FCNCs	22.4	23	.50
EWPOs	30.3	31	.49
Global	301.4	278	.14

The default smelli2.3.2 $M_W$ experimental constraint includes two input experimental values: one from ATLAS [83] and one combined measurement from CDF plus Dzero [84], whereas we use a single combined value from Ref. [4] when we include the CDF II constraint upon $M_W$. This fact explains why $n_{obs}$(EWPOs) differs by 1 between the two different $M_W$ constraint options. We also display the best-fit model parameters for each option of experimental $M_W$ constraint

We now go on to examine the pulls at each best-fit point for $t/s=-3$. We define the pull for an observable with theoretical prediction *P*, measured central value *M* and uncertainty *C* to be

4.14 $$\begin{aligned} \text {pull} = \frac{P-M}{C}, \end{aligned}$$

where *C* does *not* include correlations with other observables, but may include theoretical uncertainties added in quadrature in some cases. In particular, for $M_W$, we have added an estimated uncertainty in the prediction of 5.6 MeV [4] in quadrature.

**Fig. 2:**
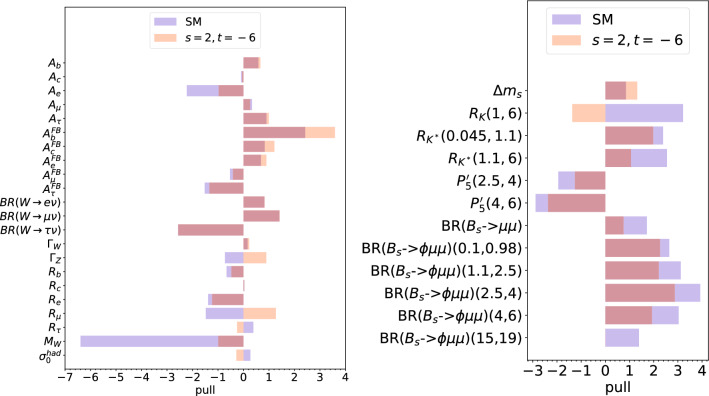
Pulls of interest including the recent CDF measurement of $M_W$ i.e. (1.1) and $n=3$. In the left-hand panel, we display the EWPOs, whereas in the right-hand panel, we display selected observables of interest to $b \rightarrow s l^+l^-$ anomalies

**Fig. 3:**
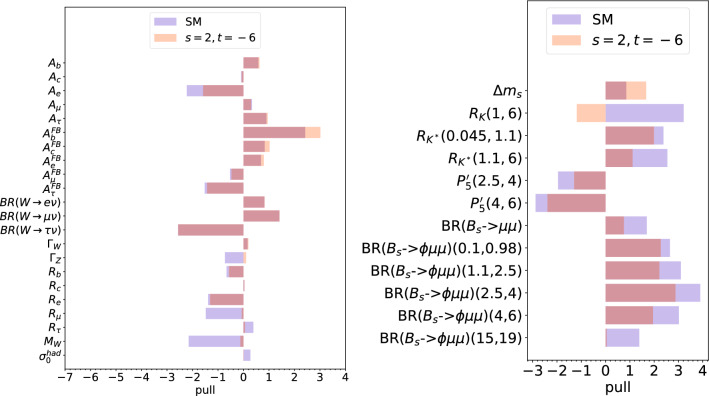
Pulls of interest for the default smelli2.3.2 constraint on $M_W$ (i.e. excluding the recent CDF measurement) and $n=3$ as in (4.13). In the left-hand panel, we display the EWPOs, whereas in the right-hand panel, we display selected observables of interest to $b \rightarrow s l^+l^-$ anomalies

We display the pulls for the combination of $M_W$ measurements that include the recent CDF II determination in Fig. 2. Several notable effects are evident: for example, unsurprisingly $M_W$ itself is better fit, with a pull of $-1$. The observable $R_\mu$, the branching ratio of the $Z^0$ boson to $\mu ^+\mu ^-$, has been increased by a fair amount; in the SM the prediction was high by over 1$\sigma$, but in the $Z^\prime$ model with $t/s=-3$ it is less than one sigma too *low*^10^. We see that the forward-backward asymmetry measured in $e^+e^-\rightarrow b \bar{b}$, $A_{FB}^b$, has a worse fit in the $Z^\prime$ model. $A_e$, the left-right asymmetry in $e^+e^-\rightarrow e^+e^-$, has a smaller pull in the $Z^\prime$ model. Overall, the quality of fit to the EWPOs is fine: Table 4 shows that the *p*-value is .29. The right-hand panel of Fig. 2 shows that many of the selected observables of interest to *b*-meson decays are fit better than in the SM save for the $B_s-\overline{B_s}$ mixing observable $\Delta m_s$, which receives (fairly mild) positive corrections from the $Z^\prime$ contribution.

We compare and contrast the fits *including* the CDF II $M_W$ measurement from Fig. 2 with fits *excluding* it in Fig. 3. The most obvious effect of excluding the CDF II $M_W$ measurement is that the SM pull of $M_W$ of is only 2$\sigma$ when the CDF II $M_W$ measurement is excluded. Because the required shift in $M_W$ is smaller, the relative effect upon the other EWPOs is smaller and the result is a good fit to EWPOs for $t/s=-3$: Table 4 reveals the *p* value in the fit to be .49. The fits in the *b*-observables on the right-hand panel show a very similar pattern between Figs. 2 and 3. Essentially, $g_X$ is being fixed by the EWPOs (and is being pulled by $M_W$ in particular), and then $\theta _{sb}$ is fit to a value which fits the $b\rightarrow s \ell \ell$ anomalies. Here, many of the SM-discrepant observables relevant to the *b*-anomalies receive a relative contribution from the $Z^\prime$ [50] $\propto g_X^2 \sin 2 \theta _{sb}/M_X^2.$ We see that the pull of $\Delta m_s$, which is a measure of the $B_s-{\overline{B}_s}$ mixing, decreases when one includes the CDF II $M_W$ measurement. This is because the fit is pushed to a larger value of $g_X$ in order to fit the larger needed new physics contribution in $M_W$. In turn, in order to fit the *b*-anomalies, one requires a smaller value of $\theta _{sb}$ in order to keep the $Z^\prime$ contributions to them (4.9) constant, in turn reducing the $Z^\prime$ contribution to $B_s-{\overline{B}_s}$ mixing.

The *p* values and pulls for the $n=2$ fits are qualitatively similar to those for $n=3$ and we neglect to present them here, noting that they are presented in the ancillary information attached to the arXiv preprint version of this paper. Excluding all of the $M_W$ measurements except for the CDF II one, the fits do not differ in significant details (the global *p* values differ by less than .02, for example) and we also relegate plots for them to the ancillary information.

## Conclusions

A $Z^\prime$ model where the SM is augmented by an additional, spontaneously broken *U*(1) gauge group can simultaneously fit both the CDF $M_W$ anomaly and the $b\rightarrow s \ell \ell$ anomalies. The model retains the other desirable properties of the $Y_3$ model on which it is based [50]: namely that it qualitatively explains a hierarchically heavy third generation of quarks and small CKM angles. As Fig. 1 demonstrates, the log likelihood contribution from CDF’s $M_W$ measurement is instrumental in picking out favoured values for the $U(1)_X$ quantum numbers of the fields. A particularly simple anomaly-free combination $Y_3 - 3 (B_3-L_3)$ has a high quality of fit (where the $\ell _3$ field is subsequently identified with the left-handed *muon* lepton doublet^11^). We note that for the combination $Y_3 - 3(B_3-L_3)$, the ‘LFU’ and ‘EWPO’ classes of observable (both of which have small theoretical uncertainties) each separately have a better quality of fit than the ‘quarks’ class, where there is more room for argument about the prediction and the size of the theoretical uncertainty assigned.

We have not developed details of the ultra-violet model, preferring instead to begin by working with an effective theory with $SU(3)\times SU(2)_L \times U(1)_Y \times U(1)_X$ gauge symmetry^12^. Light family Yukawa couplings are expected to result from some non-renormalisable operators, having integrated out heavy multi-TeV fermions that are vector-like representations of the gauge group, for example. We argue, following Ref. [86], that it is premature to set the details of the model in the ultra-violet more than we have, preferring instead to allow the data (such as the new measurement of $M_W$) to inform the model building in a more fundamental and vital way. Indeed, we have used it in the present paper to select $U(1)_X$ charges under the symmetry group.

In the coming years, the LHC experiments will provide valuable further empirical measurements of $M_W$ and observables pertinent to the $b\rightarrow s \ell \ell$ anomalies and Belle II data will also weigh in. In the meantime, an obvious avenue of interest is from direct searches for the $Z^\prime$. The most promising channels [81] are likely to be $pp \rightarrow Z^\prime \rightarrow \mu ^+ \mu ^-$ with or without additional *b*-jets. LHC and HL-LHC sensitivity estimates are an obvious target for our models for future study.^13^

## Data Availability

This manuscript has no associated data or the data will not be deposited. [Authors’ comment: This is a theoretical study and no experimental data has been listed.]

## Footnotes

1 For an electroweak fit in terms of *S*, *T* and *U* parameters, see Ref. [3].

2 We use the development version of smelli2.3.2 and its sub-program flavio2.3.3 that were on github on 27/4/22.

3 The equations for cancellation of local gauge anomalies have been solved analytically for a gauge group of the form SM$\times U(1)_X$ [37], although it is often easiest to search lists to identify anomaly-free charge assignments of phenomenological interest [38]. It has also been shown [39] that a SM$\times U(1)_X$ gauge theory suffers from no global gauge anomalies provided only that the $SU(2)_L$ anomaly [40] cancels, as it does here.

4 We ignore possible discrete quotients of the gauge group because they are not relevant to our discussion.

5 This is because all charges may be scaled by an overall factor, so long as the $U(1)_X$ gauge coupling $g_X$ is scaled by one over this factor, with no change to the physics.

6 Another route is to look for anomaly-free deformations of (2.5) in which two families of leptons are charged, generalising [52], which allow either no renormalisable lepton Yukawa couplings, or at most the $y_\tau \overline{\ell }_3^\prime H e_3^\prime$ coupling.

7 We define $c_x:=\cos x$, $s_x:=\sin x$ throughout this paper, for various quantities *x*.

8 Of course these contributions to the WET coefficients can also be derived at the level of the SMEFT matching. From this perspective, the SMEFT-WET matching formulae are [79, 80]:

4.8 $$\begin{aligned} C_9^{ij}&= \frac{4\pi ^2}{e^2} \frac{v^2}{V_{tb} V_{ts}^*} \left[ -\frac{1}{k}\left( C_{\phi q}^{(1)\, 23} + C_{\phi q}^{(3)\, 23}\right) + C_{\ell q}^{(1)\, ij23} + C_{\ell q}^{(3)\, ij23} + C_{qe}^{23ij} \right] \nonumber \\ C_{10}^{ij}&= \frac{4\pi ^2}{e^2} \frac{v^2}{V_{tb} V_{ts}^*} \left[ C_{\phi q}^{(1)\, 23} + C_{\phi q}^{(3)\, 23} - C_{\ell q}^{(1)\, ij23} - C_{\ell q}^{(3)\, ij23} + C_{qe}^{23ij}\right] . \end{aligned}$$

Substituting in the SMEFT Wilson coefficients from Table 3 reproduces (4.8).

9 Strictly speaking, we calculated the fit at a reference point of $M_X=1$ TeV.

10 Observables such as $R_\mu$ receive contributions from $(C_{\phi l}^{(1)})^{22}$, $C_{\phi e}^{22}$. As a reference to Table 3 and (2.2–2.5) shows, the sign of these contributions depends upon the sign of *t*/*s*. We have checked that the predictions for such observables do indeed go in the ‘wrong’ direction for positive *t*/*s*, resulting in a less favourable fit.

11 Identifying $e_3$ with the right-handed muon field in addition also provides a similarly acceptable fit.

12 The sub-Planckian Landau poles [85] in $g_X$ may then be mitigated by new heavy (but sub-Planckian) states originating from a larger non-abelian symmetry that is spontaneously broken to SM $\times U(1)_X$.

13 Re-casts of LHC Run II $Z^\prime$ searches for similar models (e.g. the $Y_3$ and $B_3-L_3$ models) indicate that the bounds are currently rather weak [81].
